# Characterization of immune cells in periodontitis using a new histological immunologic gingival (IG) score

**DOI:** 10.1038/s41598-025-25014-3

**Published:** 2026-01-16

**Authors:** Emilie Hascoët, Frédéric Blanchard, Marie-Astrid Boutet, Maeva Dutilleul, Jérôme Guicheux, Philippe Lesclous, Alexandra Cloitre

**Affiliations:** INSERM, Regenerative Medicine and Skeleton, RMeS, UMR 1229, Nantes Université, Oniris, Univ Angers, CHU Nantes, 44000 Nantes, France

**Keywords:** Periodontitis, Inflammation, Immunologic gingival (IG) score, Immunohistochemistry, Personalized medicine, Adaptive immunity, Infectious diseases, Inflammation, Innate immune cells, Dental diseases

## Abstract

Periodontitis is a prevalent chronic inflammatory disease due to the host response to dysbiotic biofilm. Immunohistochemistry is often used for a better and more functional tissue characterization. The main objective of this study was to create a semi-quantitative Immunologic Gingival (IG) score for characterizing immune cells in periodontitis based on five immunohistochemical stainings. The study included 11 healthy controls and 11 periodontitis patients. To determine the IG score, an atlas was drawn up with five immunohistochemical stainings (CD138, CD3, CD20, CD68, CD66b). The mean IG score was higher for periodontitis patients than for healthy controls. For each marker of the IG score, a positive correlation was found between the semi-quantitative score and the respective percentage of positive cells. An IG score of 4 out of 15 was the best cut-off for discrimination between healthy controls and periodontitis patients, with a specificity and a sensitivity exceeding 90%. The IG score provides standardized information on the immune cell signature in gingival samples from patients with periodontitis. This score could be applied in translational research but also in clinical practice, particularly for the development of personalized therapies for patients with recurrent periodontitis.

**Supplementary Information:**

The online version contains supplementary material available at 10.1038/s41598-025-25014-3.

## Introduction

Periodontitis is a multifactorial chronic inflammatory disease resulting from a dysbiotic biofilm and leading to the destruction of the alveolar bone^1^. Although dysbiosis is at the origin of the disease, the exacerbated host inflammatory immune response plays a major role in periodontal destruction and disease persistence^2^. Thus, despite well-managed mechanical treatment and rigorous oral hygiene, the recurrence rate can reach 26%^3^. Rheumatoid arthritis (RA) is a systemic autoimmune disease characterized by chronic inflammation of the joints leading to increased bone resorption and multiple joint disabilities^4^. RA and periodontitis share immunopathogenic similarities^5^. In patients with concurrent RA and periodontitis, disease-modifying antirheumatic drugs have been reported with contradictory results in term of clinical efficacy in the literature^6^. This suggests that periodontitis, like RA, is heterogeneous and presents with different profiles.

Immunohistochemistry (IHC) is often used for a better and more functional characterization of synovitis in RA. In this context, Najm et al. have developed an immunologic synovitis score (IMSYC) that can be used as a biomarker for prognosis and treatment decision-making^7^. Pitzalis et al. have described three different synovial pathotypes^8^: (1) lympho-myeloid; (2) diffuse-myeloid; and (3) pauci-immune pathotypes, strongly suspected to be associated with the clinical course of the disease, the prognosis, and also the response or resistance to treatment^9,10^.

For 30 years, several studies have focused on the composition of gingivitis and periodontitis lesions using IHC ^11–13^. However, to date, no immunohistochemical score has been described to characterize immune cells in periodontitis. Such a score would have potential applications in translational research for the study of pathogenic mechanisms of periodontitis with common immune cell signatures (mechanisms of the immune response at the cellular and molecular levels). It may lead to the development of personalized therapies in patients with refractory or recurrent disease in the future.

In this study, we created a semi-quantitative immunologic gingival (IG) score based on five immunohistochemical stainings: CD68 (macrophages), CD3 (T cells), CD20 (B cells), CD138 (plasma cells), and CD66b (neutrophils). These markers were selected on the basis of their preponderance in the tissue and their involvement in inflammation and bone resorption in periodontitis^5^.

The primary objective of this study was to develop and validate an IG score in periodontitis and to compare its diagnostic performance with the gold standard of clinical diagnosis. The secondary objective was to investigate (i) the correlations between this score and the percentage of gingival labeled cells and (ii) the correlations between the IG score and demographic, clinical, and bacteriologic data.

## Results

### Patient characteristics

Twenty-two patients were included in the study, 11 healthy controls and 11 patients with periodontitis. Their demographic, clinical, and bacteriologic characteristics are summarized in Table 1. Patients with periodontitis were significantly older than healthy controls (median age 55 ± 6 years vs. 17 ± 3.5 years, respectively). There was no significant difference in gender (female/male: 9/2 vs. 4/7) or smoking between the two groups. The difference in total bacterial count between the two groups was not significantly different. *P. gingivalis* was present in only one healthy control, whereas it was present in 10 of the 11 periodontitis patients. Thus, the amount of *P. gingivalis* and the *P. gingivalis* / total bacteria ratio were significantly higher in the periodontitis group (33 ± 40 vs. 0 ± 0, respectively, *p* < 0.001).

**Table 1:** Demographic, clinical, and bacteriologic data of included subjects.

	Healthy controls	Periodontitis patients	*p*
Age (years)	17 (17–20.5)–19	55 (52–58)–55	****
Gender, F/M	9/2	4/7	ns
Current/past smokers (*n*)	1/11	6/11	ns
Periodontitis stage	NA	4 (3–4)–3.64	****
Probing pocket depth (mm)	2 (1–2)–1.6	7 (6–8)–6.91	****
Clinical attachment loss (mm)	0	3 (3–7)–4.64	****
Bleeding on probing (%)	0	100	
Amount of total bacteria (ng)	6.93 (4.8–11.3)–7.7	11.98 (7.4–35.3)–20.22	ns
Amount of *P. gingivalis* (ng)	0 (0–0)–0.3	3.3 (1.5–7.8)–4.16	***
*P. gingivalis* / total bacteria ratio (ng)	0 (0–0)–2.6	33 (14–54)–31.9	***

Data are shown as median (Q1-Q3) - mean, Healthy controls *n* = 11, periodontitis patients *n* = 11, F: female, M: male, ns: nonsignificant, *p*-value was calculated using the Mann–Whitney U test for quantitative variables and the Fisher’s exact test for qualitative variables ***p < 0.001, ****p < 0.0001.

### Semi-quantitative scoring: IG score

**Fig. 1:**
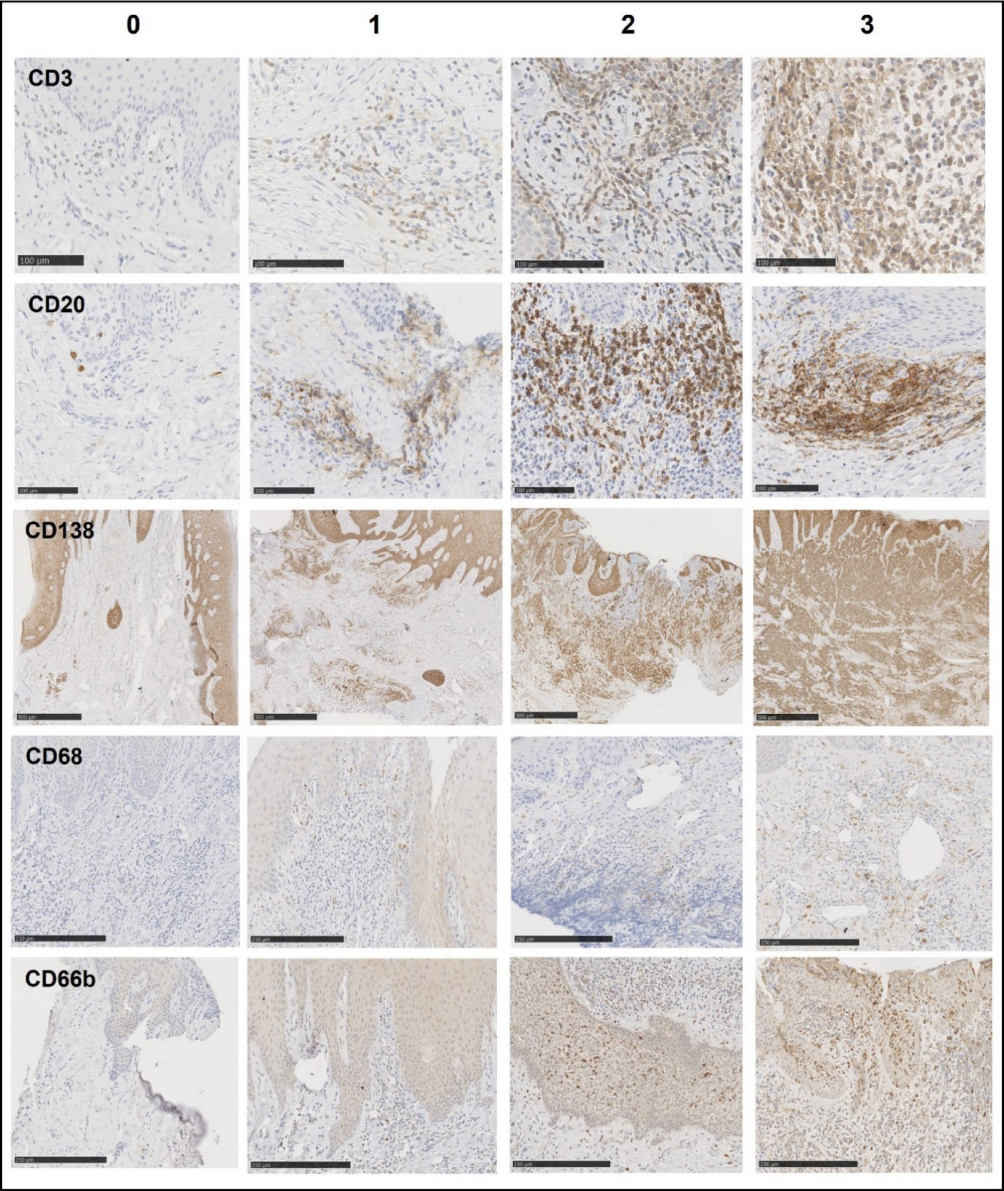
Atlas for semi-quantitative scoring of the CD3, CD20, CD138, CD68, and CD66b immunostainings to determine the immunologic gingival (IG) score**.** Each immunostaining was scored from 0 to 3 points. The total IG score was 15 points. Scale bars = 100 µm, 250 µm, and 500 µm.

The atlas for semi-quantitative scoring used to determine the IG score is shown in Fig. 1. CD3^+^ and CD20^+^ cells were found mainly at the epithelium–connective tissue junction, and 10 of the 11 periodontitis tissues had CD3^+^ and CD20^+^ cells in similar tissue areas. CD138^+^ cells were found further away from this junction in the connective tissue. CD68 cells were scattered throughout the connective tissue, while CD66b^+^ cells were mainly found at the epithelio-connective junction.

The agreement between the three observers in determining the IG score was very high, as only two (1.8%) of the 110 histologic sections analyzed had to be re-examined to reach a consensus (Supplementary Table 1).

**Fig. 2:**
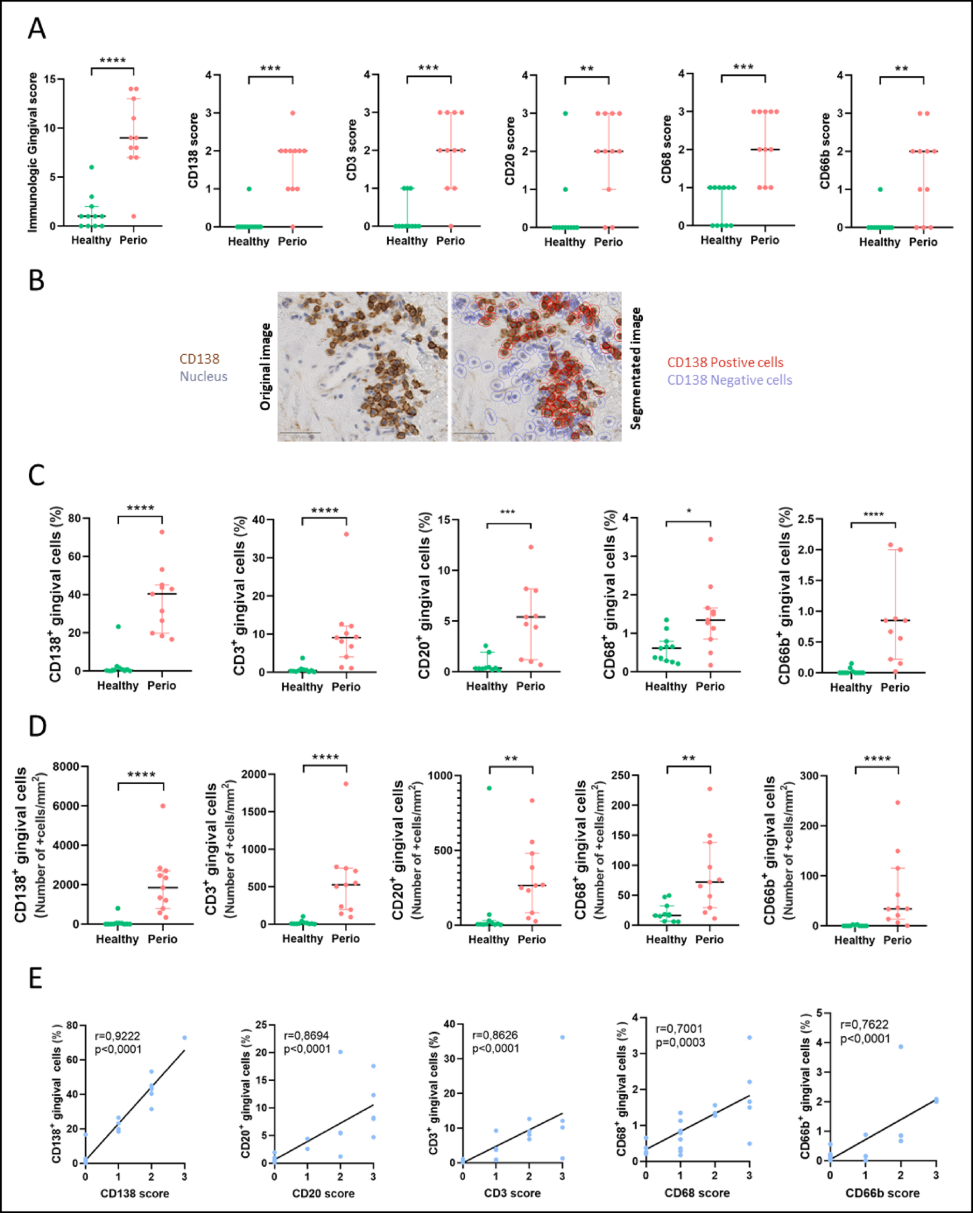
Semi-quantitative and quantitative scoring of immune cell infiltration in the gingiva of healthy controls (green circle dots) and periodontitis patients (pink circle dots). (**A**) Immunologic gingival (IG) score (0–15 points) and CD138, CD3, CD20, CD68, and CD66b semi-quantitative scores (each immunostaining: 0–3 points) in gingival samples. Individual values, median, and interquartile range are shown. (**B**) Digital image analysis was performed on gingival sections using QuPath software. Representative images of both the original immunohistochemistry (upper panels: 3,3′-diaminobenzidine (DAB)-positive cells in brown, nuclei in blue) and segmented (lower panel: DAB-positive cells are circled in red, DAB-negative cells are circled in blue) images are shown. Scale bar = 20 µm. (**C**) Percentage of CD138^+^, CD3^+^, CD20^+^, CD68^+^, and CD66b^+^ cells in gingival samples. Individual values, median, and interquartile range are shown. (**D**) Number of positive cells/mm^2^ for CD138^+^, CD3^+^, CD20^+^, CD68^+^, and CD66b^+^ cells in gingival samples. (**E**) Correlations between the percentage of CD138^+^, CD3^+^, CD20^+^, CD68^+^, and CD66b^+^ cells and the semiquantitative scores for CD138, CD3, CD20, CD68, and CD66b markers, respectively, for all patients (some points are superimposed). Individual values, median, and interquartile range are shown. *p*-value was calculated using the Mann–Whitney U test * *p* < 0.05, ** *p* < 0.01, *** *p* < 0.001, **** *p* < 0.0001.

The mean IG score was higher in tissues from patients with periodontitis than in healthy controls (9 ± 6 vs. 1 ± 2.5, respectively, *p* < 0.0001) (Fig. 2A; Supplementary Table 2). The mean score for each marker was also higher in samples from patients with periodontitis than in healthy controls (Fig. 2A): CD138 (2 ± 1 vs. 0 ± 0, *p* = 0.0001); CD3 (2 ± 2 vs. 0 ± 0, *p* = 0.0003); CD20 (2 ± 2 vs. 0 ± 0, *p* = 0.0042); CD68 (2 ± 2 vs. 1 ± 1, *p* = 0.0002); and CD66b (2 ± 2 vs. 0 ± 0, *p* = 0.0027).

### Quantitative scoring: validation of the relevance of semi-quantitative scores

To quantify the immune cells in our gingival samples, we first determined the average size of the areas analyzed per cell marker (Table 2). For four of the five IG score markers—CD138, CD20, CD68, and CD66b—the areas analyzed were significantly larger in patients with periodontitis than in healthy controls. To account for the different proportions of the areas analyzed, we next quantified immune cells by cell density in numbers/mm^2^ rather than absolute numbers.

**Table 2:** Gingival areas (expressed in mm^2^) analyzed via QuPath for CD138, CD3, CD20, CD68, and CD66b immunostainings in healthy controls and periodontitis patients.

	CD138	CD3	CD20	CD68	CD66b
Healthy controls	2.6 (1.4–3.9)–2.9	2.2 (1.2–4.4)—3.1	4.2 (2.4–6.7)–4.3	1.9 (1.2–3.7)–2.3	1.9 (1.1–3.2)–2.6
Periodontitis patients	7.1 (5.7–7.9)–6.4	4 (2.7–7.1)–4.3	7.5 (5.2–11.7)–8	3.6 (2.8–6.5)–4.4	8 (4.6–11)–7.2
*p*	**	ns	*	**	**

Data are shown as median (Q1-Q3)–mean, Healthy controls *n* = 11, periodontitis patients *n* = 11, *p*-value was calculated using the Mann–Whitney U test. ns: nonsignificant *p* value, *p < 0.05, **p < 0.01.

For each marker of the IG score, the percentage of positive cells and the number of positive cells/mm^2^ were significantly higher in samples of periodontitis patients compared with healthy controls (Fig. 2B–D). The two most abundant cell types in the gingiva of periodontitis patients were plasma cells (CD138^+^, 40.4 ± 25% of labeled cells) followed by T lymphocytes (CD3^+^, 9.1 ± 8.1% of labeled cells), with a notable dispersion of the data. Adaptive immune cells (plasma cells (CD138^+^), B cells (CD20^+^), and T cells (CD3^+^)) predominated in the periodontitis tissue (50%), whereas macrophages (CD68^+^) were poorly represented (1.3 ± 0.8%).

**Fig. 3:**
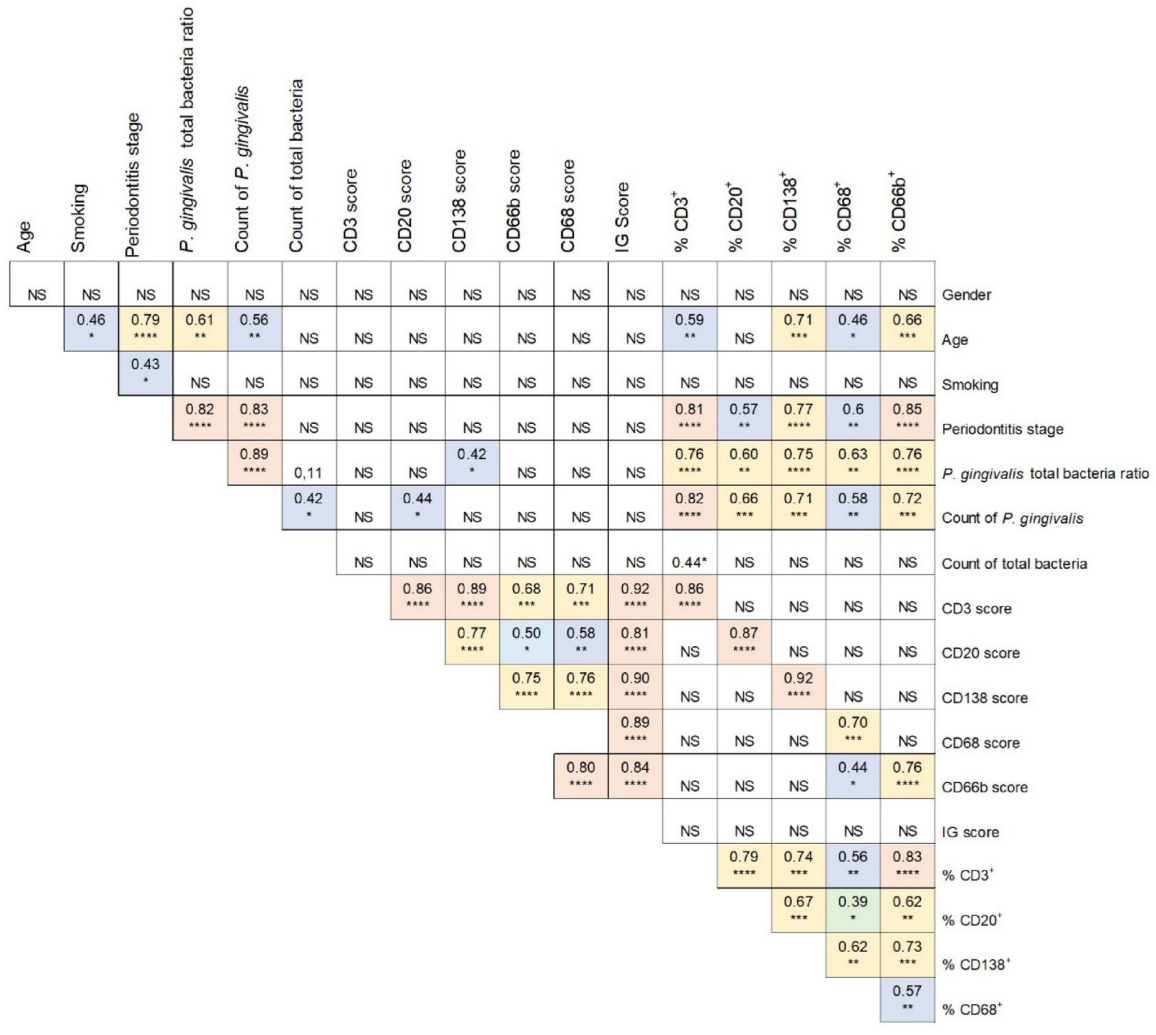
Correlations between immunologic gingival score and demographic, clinical, and bacteriologic parameters and percentage of labeled cells. Slight correlations (< 0.4) are shown in green, moderate (0.4–0.59) in blue, strong (0.6–0.79) in yellow, and very strong (> 0.8) in red. Correlations and *p*-value were evaluated by Spearman’s bivariate analysis *p < 0.05, **p < 0.01, ***p < 0.001, ****p < 0.0001. Ns: nonsignificant *p* value, IG score: immunologic gingival score.

For each marker of the IG score, a significant positive correlation was found between the semi-quantitative score and the respective percentage of positive cells (Figs. 2E and 3).

### Correlations between IG score and demographic, clinical, and bacteriologic parameters and percentage of labeled cells in gingival samples

The stage of periodontitis was positively and significantly correlated with patient age, smoking status, *P. gingivalis* count, *P. gingivalis* total bacteria ratio, and the percentages of each cell marker (Fig. 3). For each marker, the semi-quantitative score was positively and significantly correlated with the semi-quantitative score of the other markers. Similarly, there was correlation between all cell percentages. However, for each marker, there was not necessarily a significant correlation between the semi-quantitative score and the cell percentages of the other markers.

The *P. gingivalis* count correlated positively and significantly with the total bacteria count and the CD20 score as well as with all cell percentages of the IG score markers. The *P. gingivalis* total bacteria ratio was positively and significantly correlated with the CD138 score and all cell percentages of the IG score markers. As there was only one healthy control with *P. gingivalis* (*n* = 1), a subgroup analysis of patients with periodontitis was performed and also showed a strong positive and significant correlation between the amount of *P. gingivalis* and the CD20 score (0.76, *p* = 0.009).

### Diagnostic performance of the IG score

**Fig. 4:**
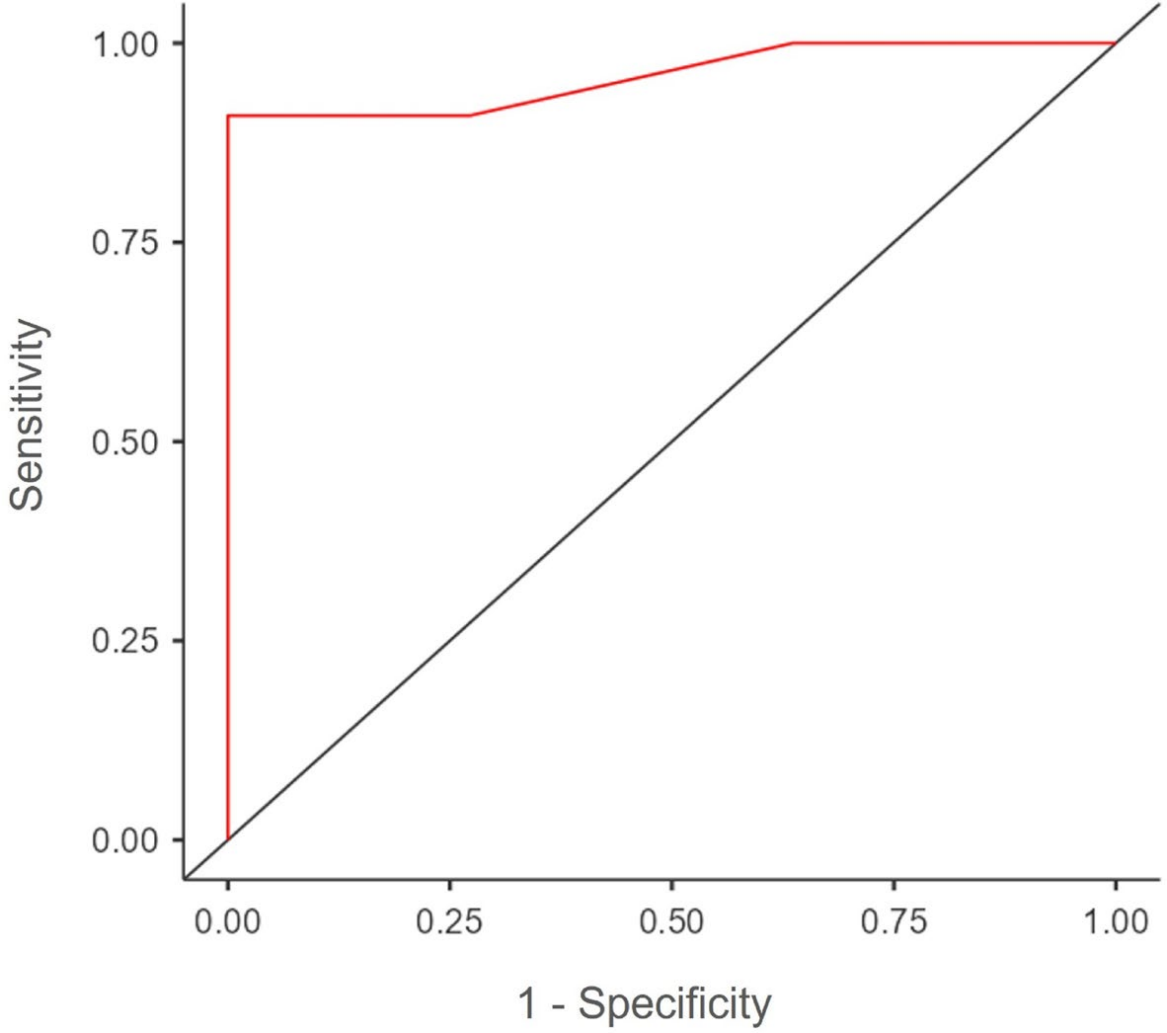
ROC curve analysis of immunologic gingival score.

ROC curve analysis of the diagnostic performance of the score determined the best cut-off for discrimination between healthy controls and periodontitis patients to be between 4 and 6, with a specificity of 90.9% and a sensitivity of 90.9% (Fig. 4). In order to limit the number of false-negative results (i.e., patients labeled as healthy controls when they had periodontitis), as a first step, the IG score threshold for discriminating healthy controls from periodontitis patients was set at 4.

## Discussion

In this study, we demonstrated that the semi-quantitative IG score, based on five immunohistochemical stainings—CD68 (macrophages), CD3 (T cells), CD20 (B cells), CD138 (plasma cells), and CD66b (neutrophils)—allowed for significant and rapid discrimination between the gingiva of healthy controls and that of patients with periodontitis. The semi-quantitative scoring method for the IG score was supported by a significant positive correlation between the grading of each cell type of the score and the corresponding percentage of positive cells obtained by quantitative analyses. A score of 4 out of 15 was the best cut-off for discriminating between healthy controls and periodontitis patients, while including the fewest false-negative results possible, with a specificity of 90.9% and a sensitivity of 90.9%.

The cellular distribution within the gingiva described herein is comparable to other IHC studies in the literature^12,13^. In single-cell RNA sequencing, Li et al. found a predominance of plasma cells^14^, whereas for William et al. T cells were the main immune cell population, as seen for healthy controls^15^. Regarding the location, the spatial transcriptomic study by Easter et al. founded the same cell distributions and proportions with the adaptive peri-sulcular immune foci^16^. This zone favored more immune–immune interactions within cell neighborhoods for innate and adaptive immune cell types, in contrast to the peri-junctional region, which favored interactions between transitional monocytes/macrophages, macrophages, and neutrophils.

In this study, patients with periodontitis had a high prevalence of plasma cells, B and T cells.CD3^+^ and CD20^+^ cells were present in the same place on the section. This profile was similar to the lympho-myeloid synovial pathotype described in RA^8^. However, one sample showed a pauci-immune profile. It will now be necessary to carry out a study examining a larger cohort of patients to validate the existence of such pathotypes in periodontitis. The difficulty of obtaining samples at each stage of the disease means that there is currently no consensus in the literature on how cell proportions change as a function of the stage and the treatment. In our study, patients with periodontitis had a greater disparity in CD3^+^ and CD138^+^ cell proportions compared with healthy controls. This may be related to the stage of periodontitis, but the patients included were fairly homogeneous, with only stage III or IV periodontitis. Other explanations could be the heterogeneity in the immune response to bacterial challenge between patients^2^ or the diversity of microbial dysbiosis. Caetano et al. have shown that the proportion of plasma cells increases from histologically healthy samples to histologically severe periodontitis samples, while memory B cells and T cells are more abundant in samples with mild periodontitis and then decrease in samples with severe periodontitis^17^. For Chen et al., B cells are the most abundant cells in patients with periodontitis and plasma cells make up the majority of cells in periodontitis patients within 1 month of initial periodontal therapy^18^. All these points demonstrate the greater heterogeneity in the immune cell profiles of periodontitis patients and suggest the importance of individualized immune cell characterization to improve the management of patients with periodontitis.

Although the immune response plays a crucial role in the development and persistence of periodontitis, microbial dysbiosis remains the trigger for the disease and is also involved in its onset^19^. In this study, *P. gingivalis* was present in only one healthy control and in 10 patients with periodontitis. This prevalence was consistent with the literature^20,21^. Easter et al. found that it was the periodontopathogen that increased the most between healthy controls and patients with periodontitis, with an almost 200-fold increase in expression^16^. The *P. gingivalis* total bacteria ratio was positively correlated with each marker as a percentage and with the CD138 score. *P. gingivalis* counts were correlated with each marker as a percentage and showed a very strong correlation with the percentage of CD3 cells; these results are consistent with those from the study by Medara et al., showing that the reduction in the proportion of *P. gingivalis* was associated with changes in memory T cells in periodontitis^22^. In this study, we showed for the first time that *P. gingivalis* levels were significantly correlated with CD20 (B cells) scores. This strengthens the link between bacterial dysbiosis, *P. gingivalis* in particular, and the development of the adaptive inflammatory response against periodontopathogens, which may explain the difficulty of treatment and the recurrence rate^3^.

The IG score that has been developed here is a rapid semi-quantitative score for easy use. According to Krenn et al., a compromise between complexity and reproducibility is necessary in order to obtain a histopathological grading of inflammatory diseases^23^. The high concordance between the IG scores of the three observers in this study suggests that the score was easy to implement and reproducible. The diagnostic performance was remarkable, with a specificity and sensitivity greater than 90%. In comparison with RA, the Najm score had a sensitivity of 71.8% and a specificity of 97.3% and the Krenn score had a sensitivity of 66.7% and a specificity of 91.9%^7,23^. To our knowledge, this is the first description of an immunohistochemical score to characterize immune cells in the gingiva of periodontitis patients.

After external validation, the IG score appears to be immediately useful for standardization purposes in research settings. It can be used to characterize gingival samples affected by periodontitis and to provide histologic evidence of the disease. However, its clinical application seems more distant, as further validation is required in larger, more diverse cohorts, including patients with varying stages of periodontitis and at different stages of treatment in order to define how the score evolves according to these parameters and to study the genetic profile of these tissues. In addition, like the scores used in RA, this score could be used to assess the homogeneity of samples and to perform molecular analyses on comparable tissues^7^. For example, a molecular comparison of a low-inflammatory gingival sample with a high-inflammatory gingival sample would mainly reflect differences in inflammation and not the relevant pathogenic genes involved in periodontitis. By grouping samples with similar immunohistological characteristics, the IG score may help decipher the pathogenesis of periodontitis.

This IG score also has potential clinical applications. It can be used in combination with the clinical and radiographic criteria for the prognosis of periodontitis. In RA, the Krenn score is positively correlated with a destructive synovitis^23^. Due to the lack of samples of stage I or II gingivitis or periodontitis in this study, it is not yet possible to conclude whether there is a correlation between the IG score and the alveolar bone destruction associated with periodontitis. Assessing the immune cell signature in the gingiva could be a key element in disease monitoring of patients with recurrent disease in order to consider therapeutic alternatives with a view to personalized therapy.

This study has unavoidable limitations. First, this is a pilot study with a limited number of patients (22 in total), but their gingival samples were well characterized demographically, clinically, and bacteriologically. The difficulty in obtaining samples from healthy controls explains why many studies, particularly those using RNAseq, have also been published with limited numbers of patients (2–10 healthy vs. 2–18 periodontitis patients)^17,18,24,25^. This preliminary study led to the creation of the IG score. To confirm these results, a future study with statistical power (with calculation of the number of subjects and the confidence interval) adapted to the score would be needed. Second, due to the limited ability to collect gingival samples from patients with gingivitis and stage I or II periodontitis, these patients were not included in this pilot study. It would be interesting to include such samples in future work to assess their IG score. Third, there was a significant age difference between the two groups. As is often the case in periodontitis-based studies ^15,24^, this age discrepancy between patients and healthy controls is explained by the surgical procedure performed to harvest healthy gingival tissue during the extraction of impacted wisdom teeth, which is most often carried out in young adults. However, there was no significant correlation between patient age, IG score, or marker score. A future study involving a larger cohort will reveal how these parameters affect each other through an adjusted analysis. Fourth, the areas analyzed for quantitative analysis were smaller in the healthy samples than in the periodontitis samples. This is consistent with the immunohistochemical analyses presented by Thorbert-Mros et al.^12^. To take account of the different proportions of the areas studied, the density of each respective cell phenotype was expressed in numbers/mm^2^. Finally, while the IG score provides histological and spatial resolution, the time-consuming and expertise to perform biopsies and their immunohistochemical processing limits its clinical use. By contrast, GCF or saliva analyses, particularly ELISA-based methods, are non-invasive, faster, and more practical in a clinical setting. However, these approaches appear to be limited because, to date, no biomarker in these oral fluids, whether used alone or in combination, has been sensitive enough to diagnose or characterize periodontitis^26^.

In conclusion, this study provides preliminary evidence of the diagnostic potential of the IG score for characterizing periodontitis in a clearly defined collection of gingival samples. Further study involving a larger cohort is now needed to confirm these results. The IG score can provide standardized information on the immune cell signature in gingival samples from patients with periodontitis. In addition to being of interest for translational research, this score could have clinical applications, particularly with regard to the development of personalized therapies for patients with recurrent disease.

## Methods

### Patients

The study was approved by the Institutional Medical Ethics Committee of Nantes University Hospital (SVTO: DC-2011–1399). All patients and/or their legal guardians gave written informed consent before surgery. All experiments were performed in accordance with the Declaration of Helsinki. The healthy control group comprised patients with impacted third-molars that required extraction. Patients with periodontitis were those with stage III or IV of the periodontal classification^1^ with teeth that needed to be extracted. The following characteristics were recorded: demographics (gender, age), medical history (diseases, medications, tobacco use), periodontal parameters (probing pocket depth (PPD), clinical attachment loss (CAL), and bleeding on probing (BOP)), and radiographic status. Healthy controls had a periodontium with no evidence of inflammation. Patient with periodontitis had PPD ≥ 4 mm, CAL ≥ 3 mm, and the presence of BOP (stages III or IV of the periodontal classification)^1^. Patients were not included in the study if they had systemic diseases that could affect periodontal health (such as diabetes, immunologic disorders, human immunodeficiency virus infection, osteoporosis, RA); if they were pregnant; if they were taking antibiotic, anti-inflammatory, or immunosuppressive therapies in the 3 months before gingival sampling; or if they were younger than 15 years or older than 65 years.

### Bacterial samples

Before tooth extractions, a paper tip was inserted tangentially into the sulcus for a defined length of 4 mm for 20 s, rubbing against the sulcus of the periodontal tooth to be extracted or the sulcus of the second molar before third-molar extraction in healthy controls. The paper tip was then cut and stored in a dry Eppendorf tube at − 80 °C.

### Gingival sample collection

Gingival papillae were harvested after a tooth extraction if excess mucosa was present. Samples were obtained from healthy controls during impacted third-molar extraction, while those from patients with periodontitis were obtained during extraction of a tooth at stage III or IV of the periodontal classification^1^. Then, the sample was fixed in 4% paraformaldehyde for 48 h before paraffin embedding.

### Immunohistochemistry

Sequential 5-µm-thick sections of synovial tissue underwent immunohistochemical staining to determine the degree of cellular infiltration by B cells (CD20 + , Dako M00755, Denmark), T cells (CD3 + , Bio-Rad MCA1477, UK), plasma cells (CD138^+^, Abcam 128,936, UK), and monocytes/macrophages (CD68 + , My BioSource 370,295, USA) and neutrophiles (CD66b^+^, Abcam 197,678. The steps are detailed in supplementary 1. Sections were digitally scanned using the Nanozoomer S210 scanner (Hamamatsu Photonics, Japan).

### IG score

The IG score was based on five immunohistochemical stainings: CD68 (macrophages), CD3 (T cells), CD20 (B cells), CD138 (plasma cells), and CD66b (neutrophils). To determine the IG score, an atlas was created, using a semi-quantitative 4-point scale (from 0 to 3, with 0 = no infiltrate, 1 = mild infiltrate, 2 = moderate infiltrate, and 3 = severe infiltrate) for the five markers as described in RA by the IMSYC^7^. Because CD138 antibody positively stains epithelial cells, the epithelium was not included in the evaluation of CD138 labeling. The total IG score was 15 points. The IG score was then determined for each gingival sample of the biocollection by three independent observers blinded to the periodontal status. For each of the five markers, the area of interest in the section was defined as being the area with the most intense staining, and the final semi-quantitative score was determined as the score agreed by at least two observers. If the score assigned by each observer was different, the slide was re-examined to reach an agreement among the three observers.

To validate the IG score, quantitative digital image analyses were also performed on all gingival samples of the biocollection to determine the percentage of CD20, CD3, CD138, CD66b, or CD68 positive cells within the tissue using QuPath software (v0.3.0.22). The size of the analyzed areas, the number of positive cells, and the percentage of labeled cells were recorded for each patient and each marker. For each marker, the results of the quantitative digital analyses were compared with those of the semi-quantitative analyses.

### DNA extraction

Before DNA extraction, bacteria were separated from the paper points by washing with 180 µL of lysis buffer + lysozyme (1 ML lysis buffer per 20 mg of lysozyme) for 60 min at 37 °C. Then, 20 µL proteinase K was added to the Eppendorf tube. After centrifugation at 1500* g* for 1 min and recovery of the supernatant, incubation was carried out for 3 h at 56 °C. Subsequently, 2 µL RNase A (10 mg/mL) was added and allowed to stand for 10 min at room temperature. Total DNA was isolated using the NucleoSpin® Tissue kit (Genomic DNA tissue Macherey–Nagel, Germany) according to the manufacturer’s instructions. The quality and concentration of the DNA were determined using a NanoDrop® 1000 spectrophotometer (Thermo Fisher Scientific, USA).

### qPCR

Total bacteria and *Porphyromonas gingivalis* (*P. gingivalis*) levels were quantified. Primers and probes were previously described in the literature^27,28^. Primer sequences related to total bacteria (5’-AAACTCAAAGGAATTGACGGGG -3’, 3’–TTGCGCTCGTTGCGGGACT–5’) and *P. gingivalis* (5’-ATAGTAGCGTGTCCGGCTTC-3’, 3’-ATCGTAGGCGGATTGGAGA-5’) were synthesized by Eurofins Scientific® (Luxembourg). Real-time polymerase chain reaction (PCR; CFX96 thermal cycler, BioRad, USA) was performed using the SYBR®Select Master Mix (Applied Biosystems, USA).

### Statistical analyses

Differences in continuous quantitative variables were evaluated using the non-parametric Mann–Whitney *U* test, and differences in qualitative variables were evaluated using Fisher’s exact test. Correlations were evaluated by Spearman’s bivariate analysis. Receptor operating curve (ROC) analysis of the diagnostic performance was carried out to determine the best cut-off of the IG score for discriminating between healthy and periodontitis patients with the best sensitivity and specificity. Data are shown as median ± interquartile range, statistical significance was set at *p* < 0.05.

## Supplementary Information

Below is the link to the electronic supplementary material.

Supplementary Material 1

## Data Availability

The datasets generated or analyzed during the current study are available from the corresponding author on reasonable request.
