# Supplementary material for: Characterization of immune cells in periodontitis using a new histological immunologic gingival (IG) score
Source: Sci Rep. 2026 Jan 16;16:2236. doi: 10.1038/s41598-025-25014-3 (PMC12816066; doi:10.1038/s41598-025-25014-3)
Supplement: Supplementary file 1 — Supplementary Material 1 [file 41598_2025_25014_MOESM1_ESM.pdf]

Supplementary 1. Full immunohistochemistry method for ginigival samples

Sequential 5-µm-thick sections were prepared using a microtome. Sections were deparaffinized in methylcyclohexane and dehydrated in alcohol and distilled water. Slides were baked at 95°C in citrate buffer pH 6 for 15 min (CD68 and CD66b slides) or for 30 min (CD3 slides) or in EDTA buffer pH 9 for 15 min (CD20 and CD138 slides), for epitope retrieval.

After allowing the slides to cool down for 15 min, they were rehydrated in a drop of PBS1X for 5 min at room temperature. A circle was drawn around the samples with an Advanced A-PAP Pen Liquid-Blocker Regular (Cosmo Bio, USA). Slides were placed in horizontal position in a humidified chamber during all incubations and washes. Endogenous peroxidases were saturated for 10 min at room temperature with 3% hydrogen peroxide. After three 5-min rinses in PBS1X, nonspecific binding sites were blocked for 20 min at room temperature using normal horse serum (Vector S-2012-50, USA) blocking solution 2.5% (CD66b, CD138, CD20, CD68 slides) or normal goat serum blocking solution (CD3 slides; Sigma-Aldrich G9023, USA). Sections were then incubated with primary antibodies for 1 h at 4°C in a humidity chamber: rabbit recombinant monoclonal human CD138 antibody (Abcam 128936, UK) (1:10,000 dilution with Animal-Free Blocker® (Vector SP-5035-100)); mouse monoclonal CD68 antibody (My BioSource 370295, USAs) (1:500 dilution with Animal-Free Blocker® (SP-5035-100, Vector)); rabbit polyclonal CD66b antibody (A197678) (1:100 dilution with PBS 0.1% BSA 0.1% Tween 20); mouse monoclonal anti-human CD20cy (Dako M00755, Denmark) (1:2000 dilution with PBS 0.1% BSA 0.1% Tween 20); rat anti-human CD3 antibody (Bio-Rad MCA1477, UK) (1:200 dilution with PBS 0.1% BSA 0.1% Tween 20**)**. After three 5-min rinses in PBS1X, the slides were incubated with secondary antibodies for 30 min at room temperature: ImmPRESS® HRP horse anti-rabbit IgG (CD66b and CD138 slides) (MP-7801-15, Vector); ImmPRESS® HRP horse anti-mouse IgG (CD20 and CD68 slides) (MP-7802-15, Vector); and ImmPRESS® HRP goat anti-rat IgG (CD3 slides) (MP-7444-15, Vector). After three 5-min rinses in PBS1X, one drop of DAB (ImmPACT® DAB Substrate Kit, SK-4105, Vector) was applied to each slide for 3 min for revelation. The slides were then rinsed in a distilled water bath for 5 min. Counterstaining was carried out with Mayer's hematoxylin solution and lithium carbonate. After dehydration in alcohol and methylcyclohexane, the slides were mounted using Eukitt medium (Dutscher, France). The sections were digitally scanned using the Nanozoomer S210 scanner (Hamamatsu Photonics, Japan). The negative control was analyzed following the same procedure but omitting the primary antibody. The specificity of

the primary antibodies was verified on human spleen sections for CD3, CD20, CD68, and CD138, and on human bone marrow sections for CD66b.

Supplementary Table 1. Score per marker of the 110 histological slides and IG score assigned by each observer.

observer 1						
Patients	Score per marker					IG score
	CD3	CD20	CD138	CD68	CD66b	
1	1	0	2	3	2	8
2	0	0	0	1	0	1
3	2	2	2	1	0	7
4	3	3	1	2	1	10
5	1	3	1	1	0	6
6	0	0	0	0	0	0
7	0	0	0	0	0	0
8	2	3	2	1	1	9
9	3	3	2	3	2	13
10	1	1	1	3	2	8
11	3	3	2	3	3	14
12	0	0	0	1	0	1
13	2	1	3	3	3	12
14	0	0	0	0	0	0
15	1	1	0	1	0	3
16	0	0	0	0	0	0
17	0	0	0	1	0	1
18	0	0	0	1	0	1
19	0	0	0	1	1	2
20	2	2	1	1	0	6
21	3	2	2	2	2	11
22	1	0	0	0	0	1

The scores of the two slides that varied between observers and that were re-examined are highlighted in orange. IG score: immunologic gingival score

Supplementary Table 1. Score per marker of the 110 histological slides and IG score assigned by each observer.

Observer 2						
Patients	Score per marker					IG score
	CD3	CD20	CD138	CD68	CD66b	
1	1	0	2	3	2	8
2	0	0	0	0	1	1
3	2	1	1	1	0	5
4	1	3	1	2	1	8
5	1	3	1	1	0	6
6	0	0	0	0	0	0
7	0	0	0	0	0	0
8	2	2	1	1	1	7
9	3	3	2	3	2	13
10	1	1	1	3	2	8
11	3	2	2	3	3	13
12	0	0	0	1	0	1
13	3	1	3	3	3	13
14	0	0	0	0	0	0
15	0	1	0	1	0	2
16	0	0	0	0	0	0
17	0	0	0	1	0	1
18	0	0	0	1	0	1
19	1	0	0	1	1	3
20	2	2	1	3	0	8
21	3	2	2	2	1	10
22	1	0	0	0	0	1

The scores of the two slides that varied between observers and that were re-examined are highlighted in orange. IG score: immunologic gingival score

Supplementary Table 1. Score per marker of the 110 histological slides and IG score assigned by each observer.

Observer 3						
Patients	Score per marker					IG score
	CD3	CD20	CD138	CD68	CD66b	
1	1	1	2	3	2	9
2	1	1	0	1	0	3
3	2	2	2	1	1	8
4	2	3	1	2	1	9
5	1	3	1	2	0	7
6	0	0	0	0	0	0
7	0	0	0	1	1	2
8	3	3	2	1	1	10
9	3	3	2	3	3	14
10	1	2	2	3	2	10
11	2	3	2	1	3	11
12	0	0	0	2	0	2
13	3	3	3	3	3	15
14	1	0	0	0	0	1
15	1	1	0	2	1	5
16	0	0	0	0	0	0
17	0	0	0	1	0	1
18	0	0	0	1	0	1
19	0	0	0	1	1	2
20	1	3	1	2	0	7
21	3	3	2	1	2	11
22	1	0	0	1	1	3

The scores of the two slides that varied between observers and that were re-examined are highlighted in orange. IG score: immunologic gingival score

Supplementary Table 2. Final score per marker and IG score of healthy and periodontitis patients. IG score: immunologic gingival score

Patients		Score per marker					IG score
		CD3	CD20	CD138	CD68	CD66b	
1	Periodontitis	1	0	2	3	2	8
2	Periodontitis	0	0	0	1	0	1
3	Periodontitis	2	2	2	1	0	7
4	Periodontitis	2	3	1	2	1	9
5	Healthy	1	3	1	1	0	6
6	Healthy	0	0	0	0	0	0
7	Healthy	0	0	0	0	0	0
8	Periodontitis	2	3	2	1	1	9
9	Periodontitis	3	3	2	3	2	13
10	Periodontitis	1	1	1	3	2	8
11	Periodontitis	3	3	2	3	3	14
12	Healthy	0	0	0	1	0	1
13	Periodontitis	3	2	3	3	3	14
14	Healthy	0	0	0	0	0	0
15	Healthy	1	1	0	1	0	3
16	Healthy	0	0	0	0	0	0
17	Healthy	0	0	0	1	0	1
18	Healthy	0	0	0	1	0	1
19	Healthy	0	0	0	1	1	2
20	Periodontitis	2	2	1	2	0	7
21	Periodontitis	3	2	2	2	2	11
22	Healthy	1	0	0	0	0	1
